# Expanding the Evidence-base for Clinician-Graded Dysphagia Using Dynamic Imaging Grade of Swallowing Toxicity (DIGEST): Validation Across Non-head and Neck Cancer Oncology Populations

**DOI:** 10.1007/s00455-025-10875-7

**Published:** 2026-03-18

**Authors:** Beatrice Manduchi, Carla L. Warneke, Xiaohui Tang, Sheila Buoy, Carly E. A. Barbon, Katherine A. Hutcheson

**Affiliations:** 1 School of Public Health, The University of Texas Health Science Center at Houston, Houston, TX USA; 2 Department of Head and Neck Surgery, The University of Texas MD Anderson Cancer Center, Houston, TX USA; 3 Department of Biostatistics, The University of Texas MD Anderson Cancer Center, Houston, TX USA; 4 Division of Radiation Oncology, The University of Texas MD Anderson Cancer Center, Houston, TX USA

**Keywords:** Videofluoroscopy, Neuro-oncology, Thoracic, Endocrine, Thyroid, DIGEST

## Abstract

**Abstract:**

The Dynamic Imaging Grade of Swallowing Toxicity version 2 (DIGEST_v2_) is a clinician-graded scale for assessing pharyngeal dysphagia in modified barium swallow (MBS) studies, initially validated for head and neck cancer (HNC) patients. Given the investigators’ published clinical application more broadly in all oncology populations in a comprehensive cancer center, this study evaluated the validity of DIGEST_v2_ across diverse non-HNC oncology populations. A retrospective analysis included 386 patients who underwent MBS studies at MD Anderson Cancer Center (2016–2021). Participants were randomly selected to represent a mix of the most common referrals for MBS (neuro-oncology, endocrine/thyroid, thoracic cancers, mixed cancer etiologies in both inpatient and outpatient care). Two independent raters, clinical and research, assigned DIGEST overall, safety, and efficiency grades. Inter-rater reliability was assessed using weighted kappa (κ_w_). Criterion validity was evaluated against the MBSImP pharyngeal total score, and convergent construct validity against the MDADI physical score and the PSS-HN diet score, using Spearman’s correlation coefficients (r_s_) and Kruskal-Wallis tests. Analyses were stratified by disease and setting subgroups. Inter-rater reliability for the overall sample and subgroups was substantial for DIGEST overall, safety, and efficiency grades (κ_w_ = 0.68–0.71), with moderate agreement for efficiency in the endocrine/thyroid subgroup (κ_w_ = 0.55). DIGEST effectively differentiated levels of pharyngeal pathophysiology for the overall sample and subgroups (MBSImP: r_s_=0.68–0.86, *p* < 0.05), demonstrating criterion validity. Associations with MDADI physical (r_s_=-0.12 to -0.47) and PSS-HN diet (r_s_=-0.23 to -0.49) indicated weak to moderate convergent construct validity. DIGEST demonstrated robust psychometric properties for assessing the severity of pharyngeal dysphagia across diverse non-HNC cancer populations, maintaining stable performance in common subgroups referred for MBS studies. This validation supports the broader use of DIGEST in oncology practice, addressing the need for reliable dysphagia assessment tools in oncology.

**Key Points:**

- DIGESTv2 demonstrated substantial inter-rater reliability (κw = 0.68–0.71) between clinicians in real-world practice and research laboratory image grading across neuro-oncology, endocrine/thyroid, and thoracic cancer populations using routine clinical imaging data.

- Criterion validity was confirmed by strong correlations with reference measures of swallowing function (rs = 0.68–0.86), validating DIGEST’s ability to differentiate dysphagia severity.

- DIGESTv2 performed consistently across subgroups commonly referred for videofluoroscopic-based swallowing evaluations, supporting its use as a standardized assessment method in oncology care.

**Supplementary Information:**

The online version contains supplementary material available at 10.1007/s00455-025-10875-7.

## Introduction

The Dynamic Imaging Grade of Swallowing Toxicity version 2 (DIGEST_v2_) is a clinician-reported tool designed to grade the severity of pharyngeal dysphagia through videofluoroscopy, also referred to as modified barium swallow (MBS) studies [1, 2]. Using a simple flowsheet and rubric as a decision tree, DIGEST summarizes patterns of penetration/aspiration and pharyngeal residue, yielding an overall grade (0 through 4) of pharyngeal dysphagia, aligned with the National Cancer Institute’s Common Terminology Criteria for Adverse Events (CTCAE) toxicity framework [1]. Initially developed and validated for individuals diagnosed with head and neck cancer (HNC), DIGEST has shown robust psychometric properties within this target demographic, including high intra- and inter-rater reliability, criterion and construct validity [2], responsiveness to change following radiation therapy (RT) [3], head and neck surgery [4] and swallowing rehabilitation [5], and association with RT dose to swallowing muscles [3].

Since its development in 2016, DIGEST has been steadily adopted in various clinical and research settings as an evidence-based dysphagia assessment tool. For instance, at the originating site (MD Anderson Cancer Center), DIGEST has been routinely used in over 90% of clinical MBS studies conducted at the comprehensive cancer center since the tool’s development. In a recently published implementation evaluation comprising 13,055 assessments conducted by 30 speech-language pathologists (SLPs), DIGEST was used consistently across diverse oncology populations, including both HNC and non-HNC patients [6]. Additionally, translation initiatives, and published reports of real-world applications further reinforce the practical utility of DIGEST in diverse HNC clinical settings [7–9] and non-HNC patient populations [10, 11]. Building on this foundation, recent validation efforts have demonstrated DIGEST_v2_’s strong psychometric properties (including reliability, criterion and construct validity) in individuals with amyotrophic lateral sclerosis (ALS) [12] and in its adapted version for fiberoptic endoscopic evaluation of swallowing (DIGEST-FEES) to address additional clinical needs [13].

Expanding validation efforts to encompass the broader oncology population is a critical next step. Recent data from our cancer institution show that over a five-year period (2016–2021), only half of the MBS studies were conducted for HNC patients, with remaining referrals encompassing other cancer types, primarily endocrine, thoracic, hematologic, and central nervous system (CNS) malignancies [6]. This observation aligns with existing literature demonstrating a high prevalence of dysphagia in solid tumors outside anatomic swallow regions (i.e., oral cavity, pharynx, larynx, and upper esophageal sphincter) [14], with an overall 19% prevalence throughout the cancer trajectory [15]. More specifically, in lung cancer, moderate dysphagia affects 30–51% of patients during acute treatment phases, with 11–13% experiencing severe dysphagia [16, 17]. Among patients with advanced lung cancer receiving palliative chemotherapy, prevalence estimates reach 18% [18]. Similarly, CNS malignancies, whether primary brain tumors or metastatic disease, are associated with dysphagia rates of 15–26%, often attributed to sensorimotor or cognitive impairments [19–21]. In these populations, dysphagia is strongly associated with cachexia, accelerated weight loss, functional decline, and diminished quality of life [15, 22, 23]. The pathophysiological mechanisms underlying dysphagia in these cancers may differ significantly from those observed in HNC [14], necessitating the specific validation of assessment tools originally developed for HNC before their application in these groups. This underscores the critical need for effective assessment tools like DIGEST in non-HNC populations, already identified as a research priority [22] in the absence of other validated diagnostic instruments [24]. Having already demonstrated its feasibility and wide applicability in oncology practice [6], it is imperative to validate DIGEST specifically for cancers beyond the head and neck to ensure equitable and comprehensive diagnostics and care for all cancer survivors affected by dysphagia.

The current study seeks to evaluate the psychometric properties of DIGEST_v2_ in oncology populations that represent the most common non-HNC referrals for MBS, including neuro-oncology, endocrine/thyroid, thoracic cancers, and mixed cancer etiologies in both inpatient and outpatient care. This research is part of a broader clinical implementation initiative (NIH/NCI: R01CA271223) aimed at facilitating the practical adoption of DIGEST_v2_ across diverse clinical settings in oncology. We hypothesize that DIGEST will demonstrate strong reliability, as well as criterion and construct validity, across a wide range of oncology populations.

## Materials and Methods

### Sample Selection

This is a retrospective analysis (IRB: MD Anderson PA19-0261) of clinical MBS studies completed between 2016 and 2021 at MD Anderson Cancer Center (Houston, Texas). As part of this protocol, MBS examinations were conducted in patients with diagnosed or suspected cancer for one of the indications listed in Table 1.

Participants were randomly selected from this dataset to represent a mix of all non-HNC cancer types with purposive sampling to ensure adequate power (target *n* = 80 each) for 5 pre-specified subgroup analyses of interest representing the most common non-HNC referrals for MBS: neuro-oncology, endocrine/thyroid cancer, thoracic cancer, as well as mixed cancer etiology inpatient and outpatient care. Patients were excluded if they had a HNC diagnosis (including multiple cancers with HNC), had a missing diagnosis in the tumor registry, or were missing DIGEST grade in EHR record. Although thyroid malignancies are often managed by head and neck surgeons, they were classified as endocrine cancers due to their origin in hormone-producing glands. HNCs were specifically defined as cancers of the upper aerodigestive tract including paranasal sinuses, nasal cavity, oral cavity, tongue, salivary glands, larynx, nasopharynx, oropharynx, and hypopharynx [25].

### MBS Acquisition

MBS studies were acquired according to a standard protocol, including 2 trials each of 5-mL, 10-mL, and self-administered sips from a cup of thin liquid barium (Varibar; Bracco Diagnostics, Inc; IDDSI equivalent 0 [26]), barium pudding (Varibar; Bracco Diagnostics, Inc; IDDSI equivalent 4), and a cracker coated in barium pudding (IDDSI equivalent 7); thickened liquids were administered at clinician discretion and per tool instructions included in DIGEST grading when given. MBS images were recorded at 30 frames per second with audio synchronization (TIMS Medical, Foresight Imaging, Chelmsford, Massachusetts).

### Validation Measures

Each MBS study was evaluated by two independent raters: one of 19 clinical SLPs who graded DIGEST_v2_ as part of routine care and documented DIGEST grades in the EHR, and one of 5 research SLPs who were previously trained to meet DIGEST reliability standards (> 80% exact agreement on each DIGEST component with gold standard rater, i.e., DIGEST developer). Research raters were blinded to examination details and prior ratings. For each study, raters assigned DIGEST, DIGEST safety (DIGEST-S), and DIGEST efficiency (DIGEST-E) grades. DIGEST-S is determined based on patterns of airway invasion according to the Penetration-Aspiration Scale (PAS) [27], while DIGEST-E is derived from ordinal estimations of pharyngeal residue. The interaction of these grades generates the overall DIGEST grade, ranging from 0 (no dysphagia) to 4 (life-threatening dysphagia). Inter-rater reliability was assessed between the clinical and research raters’ scores.

As per the HNC validation study, the criterion validity endpoint was the MBSImP™© pharyngeal impairment total score [28]. This score, which sums 10 components of pharyngeal swallow physiology rated on an ordinal scale, indicates greater impairment with higher scores. Component 13 (assessing pharyngeal contraction in the antero-posterior view) was excluded from this analysis due to 22.5% missing data, resulting in a pharyngeal total score range of 0–26 [28]. The MBSImP pharyngeal score was also used to assess the ordinality of DIGEST in this analysis. A sensitivity analysis included Component 13, expanding the possible score range to 0–29 (*n* = 309).

Reference measures for convergent construct validity included the MD Anderson Dysphagia Inventory (MDADI) Physical subscale and the Performance Status Scale for Head and Neck Cancer Patients (PSS-HN) Normalcy of diet subscale. The MDADI is a patient-reported questionnaire assessing swallowing-related quality of life [29]. Its Physical subscale, composed of 8 items, measures the perceived physical impairment of swallowing, and its total ‘composite’ score is normalized to range from 20 (extremely low functioning) to 100 (high functioning) [29]. Finally, the PSS-HN Normalcy of diet is a clinician-rated tool indicating the patient’s level of oral intake on an 11-point scale, ranging from 0 (no oral intake) to 100 (regular oral diet) [30].

### Statistical Analysis

Sample demographics, clinical characteristics, and distributions of validation measures were summarized using descriptive statistics. Each psychometric analysis was conducted for the total sample and separately by subgroup (disease group or inpatient/outpatient setting), with DIGEST grade as the primary endpoint, and DIGEST-S and DIGEST-E grades as secondary endpoints.

Weighted k (radical weighting: 1, 0.50, 0.29, 0.13, 0) was calculated to assess the reliability of clinician- versus research-derived ratings, with k > 0.60 indicating substantial agreement [31]. Spearman correlation coefficients (r_s_) with 95% confidence intervals (CI) were computed to assess the criterion validity of DIGEST in reference to MBSImP pharyngeal score (hypothesis: strong positive monotonic association, r_s_ >0.7) and construct validity in reference to the MDADI Physical subscale and PSS-HN Normalcy of diet subscale (hypotheses: moderate negative monotonic association, − 0.4 < r_s_ < − 0.7) [32]. The Kruskal-Wallis test examined the relationship between the MBSImP pharyngeal score and DIGEST grades. For statistically significant Kruskal-Wallis tests, post-hoc pairwise comparisons were conducted to identify which DIGEST grades had statistically significant differences in MBSImp pharyngeal scores (i.e., grade 0 vs. 1 vs. 2 vs. 3 vs. 4). The Dwass-Steel-Critchlow-Fligner test was used to adjust for multiple pairwise comparisons [33]. P-values < 0.05 were considered statistically significant, and all analyses were conducted using R version 4.4.1 and Stata version 14.2 (StataCorp).

## Results

### Sample Characteristics

Of the 400 patients initially sampled, 14 were excluded due to missing DIGEST data, resulting in 386 patients included in the study. Demographic and clinical characteristics are summarized in Table 1. The mean age was 61.1 years (SD 16.3), with a majority being male (60.9%). Most MBS indications were due to patients showing dysphagia symptoms (84.9%). Most patients (25.2%) were on a full diet with no restriction (PSS-HN: 100), followed by 24.5% on a restricted solids diet (PSS-HN: 50–80), and 20.6% on a non-oral diet (PSS-HN: 0) (Table 1). Cancer diagnosis among mixed etiology subgroups is shown in Table 2.

**Table 1:** Sample characteristics (*n* = 386)

Characteristics	Value
Age at MBS, mean (SD), y	61.6 (16.3)
Sex, No. (%)	
Male	235 (60.9%)
Female	151 (39.1%)
Major disease sites or practice setting subgroup, No. (%)	
CNS	75 (19.4%)
Endocrine	77 (19.9%)
Thoracic	76 (19.7%)
Inpatient, mixed cancer etiology	79 (20.5%)
Outpatient, mixed cancer etiology	79 (20.5%)
MBS indication No. (%)	
Baseline MBS	14 (3.7%)
Rule out leak	14 (3.7%)
Symptomatic	325 (84.9%)
Swallow surveillance pathway	30 (7.8%)
MBSImP Pharyngeal score, median (IQR; range)	9.0 (6.0; 2–21)
MDADI Physical score, median (IQR; range)	62.5 (30.0; 22.5–100)
PSS-HN Normalcy of diet, No. (%)	
Non-oral (0)	58 (20.6%)
Liquid (10–20)	18 (6.4%)
Pureed/non/chewable (30–40)	23 (8.2%)
Restricted solids (50–80)	69 (24.5%)
Full with liquid assist (90)	43 (15.2%)
Full with no restriction (100)	71 (25.2%)
DIGEST grade	
0	79 (20.5%)
1	82 (21.2%)
2	88 (22.8%)
3	105 (27.2%)
4	32 (8.3%)
DIGEST-S grade	
0	124 (32.1%)
1	57 (14.8%)
2	97 (25.1%)
3	84 (21.8%)
4	24 (6.2%)
DIGEST-E grade	
0	132 (34.2%)
1	117 (30.3%)
2	25 (6.5%)
3	85 (22.0%)
4	27 (7.0%)

CNS = central nervous system; DIGEST = Dynamic Imaging Grade of Swallowing Toxicity; DIGEST-E = DIGEST Efficiency; DIGEST-S = DIGEST Safety; MBS = modified barium swallow; MBSImP = Modified Barium Swallow Impairment Profile; MDADI = MD Anderson Dysphagia Inventory; PSS-HN = Performance Status Scale for Head and Neck Cancer Patients

**Table 2:** Cancer diagnosis among the inpatient and outpatient mixed etiology subgroups (*n* = 158)

Cancer Diagnosis	Inpatient No. (%)	Outpatient No. (%)
Blood	12 (15.2%)	11 (13.9%)
CNS	12 (15.2%)	3 (3.8%)
Endocrine/Thyroid	2 (2.5%)	12 (15.2%)
GI	6 (7.6%)	1 (1.3%)
Metastatic	1 (1.3%)	13 (16.5%)
Multiple (non-HN)	15 (19.0%)	13 (16.5%)
Other solid tumors	12 (15.2%)	19 (24.1%)
Thoracic	19 (24.1%)	7 (8.9%)

CNS = central nervous system; GI = gastrointestinal; HN = head and neck

Within the total sample, DIGEST = 0 (no dysphagia) was observed in 79 patients (20.5%); DIGEST = 1 (mild dysphagia) in 82 patients (21.2%); DIGEST = 2 (moderate dysphagia) in 88 patients (22.8%); DIGEST = 3 (severe dysphagia) in 105 patients (27.2%); and DIGEST = 4 (life-threatening dysphagia) in 32 patients (8.3%) (Table 1). The distribution of DIGEST, DIGEST-S and DIGEST-E grades by subgroup is presented in eFigures 1–3 in the Supplementary Information.

### Reliability

For the total sample, inter-rater reliability between clinical and research scores was substantial for DIGEST, DIGEST-S and DIGEST-E (κ_w_ = 0.68–0.71, *p* < 0.001), as shown in Table 3. Substantial agreement was also observed for DIGEST grades in the subgroup analyses (κ_w_ = 0.62–0.79, p = *p* < 0.001), except for the endocrine/thyroid cancer subgroup, which showed moderate reliability (κ_w_ = 0.55, 95% CI 0.40 to 0.69, *p* < 0.001) for DIGEST-E (Table 3).

**Table 3:** Inter-rater reliability (weighted κ) for DIGEST grades

	DIGEST		DIGEST-S		DIGEST-E	
**Group (N)**	κ_w_	**95% CI**	κ_w_	**95% CI**	κ_w_	**95% CI**
Total sample (386)	0.70	0.66–0.75	0.71	0.66–0.76	0.68	0.63–0.73
Subgroup						
CNS (75)	0.68	0.56–0.79	0.64	0.53–0.76	0.69	0.57–0.81
Endocrine/thyroid (77)	0.77	0.68–0.87	0.77	0.70–0.86	0.55	0.40–0.69
Thoracic (76)	0.62	0.51–0.74	0.67	0.55–0.80	0.74	0.61–0.86
Inpatient (79)	0.69	0.59–0.80	0.66	0.55–0.79	0.69	0.57–0.80
Outpatient (79)	0.75	0.65–0.85	0.79	0.69–0.88	0.72	0.61–0.82

CI = confidence interval; CNS = central nervous system; DIGEST = Dynamic Imaging Grade of Swallowing Toxicity; DIGEST-E = DIGEST Efficiency; DIGEST-S = DIGEST Safety; κ_w_ = weighted kappa using radical weights

### Criterion Validity and Ordinality

**Fig. 1:**
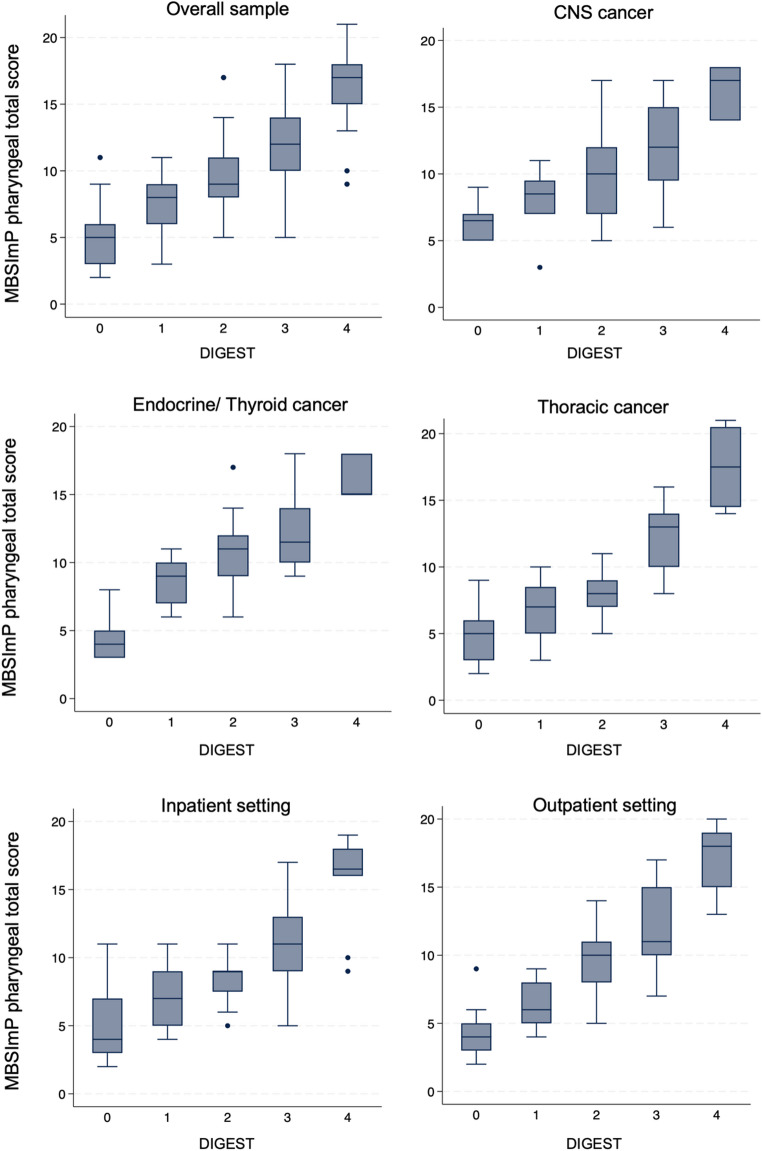
DIGEST grade according to MBSImP pharyngeal total score. DIGEST distinguished levels of pharyngeal pathophysiology in the total sample (*n* = 378; r_s_=0.78, 95% CI: 0.74–0.86), and in the subgroups of CNS cancer (*n* = 72; r_s_=0.68, 95% CI: 0.54–0.82), endocrine/thyroid cancer (*n* = 76; r_s_=0.76, 95% CI: 0.65–0.87), thoracic cancer (*n* = 73; *r* = 0.79, 95% CI: 0.69–0.90), inpatient setting (*n* = 79; r_s_=0.76, 95% CI: 0.64–0.88) and outpatient setting (*n* = 78; r_s_=0.86, 95% CI: 0.79–0.92). CI = Confidence interval; CNS = central nervous system; DIGEST = Dynamic Imaging Grade of Swallowing Toxicity; MBSImP = Modified Barium Swallow Impairment Profile

DIGEST demonstrated criterion validity as evidenced by discriminating levels of pharyngeal pathophysiology (per MBSImP pharyngeal score) for total sample (r_s_=0.78, *p* < 0.001) and across subgroups (r_s_=0.68–0.86, *p* < 0.001), as depicted in Fig. 1. Post-hoc pairwise comparisons between DIGEST and MBSImP showed statistically significant differences between all adjacent DIGEST grades (0 vs. 1, 1 vs. 2, 2 vs. 3, and 3 vs. 4; each *p* < 0.001) after adjustments for multiple comparisons and reflected ordinality of the metric for the total sample, with MBSImp pharyngeal scores (median [IQR]) increasing with increasing DIGEST scores: 5 (3), 8 (3), 9 (3), 12 (4) and 17 (3) for DIGEST grades 0, 1, 2, 3, and 4, respectively.

The sensitivity analysis including Component 13 (pharyngeal contraction in antero-posterior view) in the MBSImP pharyngeal total score showed similar results for criterion validity and ordinality as demonstrated in eFigure 4 in the Supplementary Information.

### Construct Validity

**Fig. 2:**
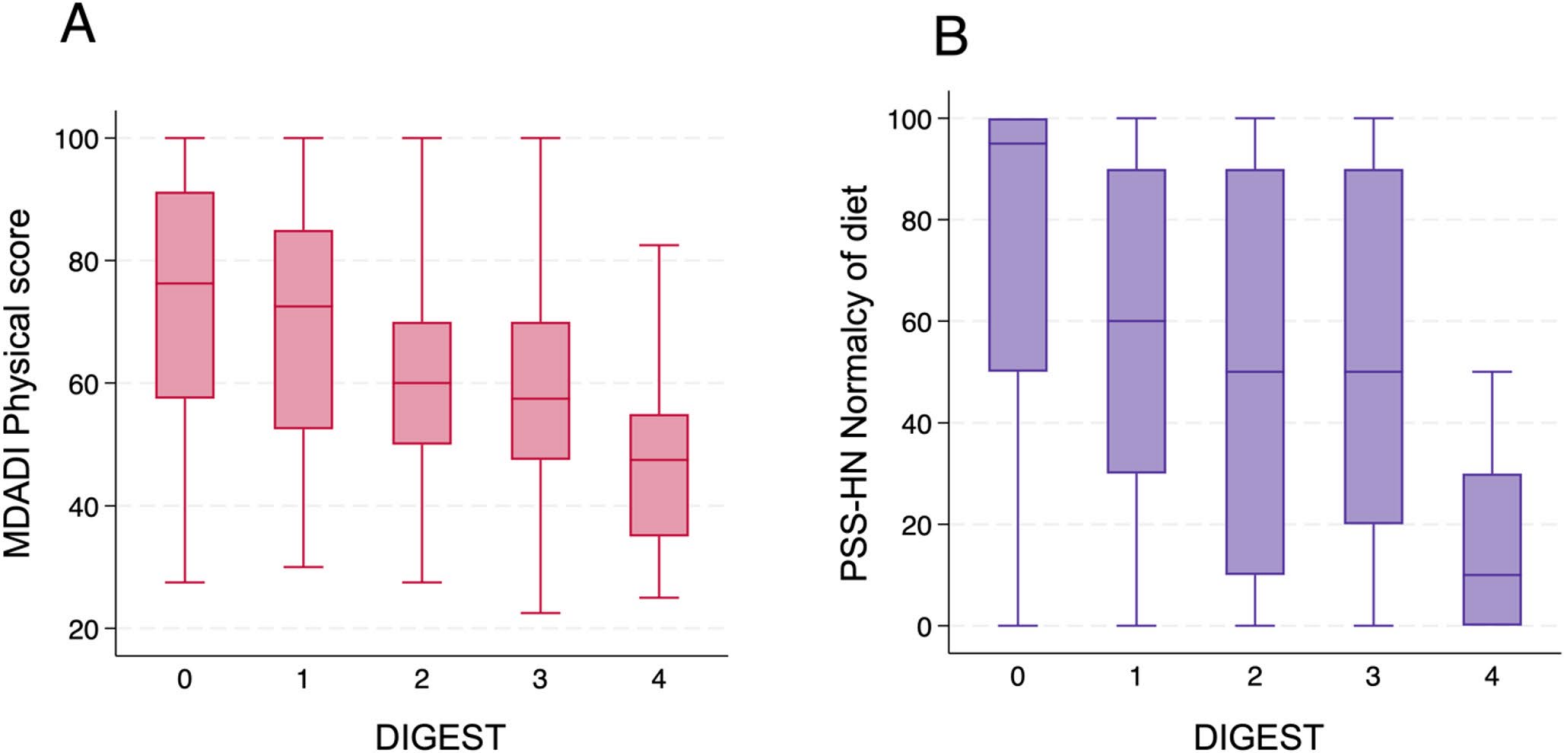
DIGEST grade according to (A) MDADI Physical score and (B) PSS-HN Diet for the total sample. DIGEST showed fair significant correlations with related constructs of perceived dysphagia (MDADI Physical score: *n* = 203; rs= − 0.36, 95% CI: − 0.48; − 0.24), and oral intake (PSS-HN diet: *n* = 282; rs= − 0.32; 95% CI: − 0.43; − 0.21). CI = Confidence interval; DIGEST = Dynamic Imaging Grade of Swallowing Toxicity; MDADI = MD Anderson Dysphagia Inventory; PSS-HN = Performance Status Scale for Head and Neck Cancer Patients

In the total sample, DIGEST demonstrated weak negative correlations with related constructs of perceived dysphagia (MDADI Physical score: r_s_=–0.36; 95% CI − 0.48, − 0.24; *p* < 0.001), and oral intake (PSS-HN diet: r_s_=–0.32; 95% CI − 0.43, − 0.21; *p* < 0.001) (Fig. 2A, B). Within individual subgroups, the associations with the MDADI were weak to moderate and statistically significant (r_s_=–0.35 to − 0.47; *p* < 0.05), except for the thoracic and inpatient subgroups, (r_s_= − 0.13 and − 0.41, respectively (*p* > 0.05). In contrast, the associations with the PSS-HN diet were moderate and statistically significant only for the thoracic and outpatient subgroups (r_s_ = − 0.41 and − 0.49, respectively; *p* < 0.001).

## Discussion

Dysphagia is a prevalent and impactful condition among cancer survivors, yet evidence-based diagnostic tools like DIGEST have primarily been validated for HNC, leaving a gap in care for patients with other cancer types [15, 24]. The current study addressed this need by evaluating the psychometric properties of DIGEST_v2_ in a sample representing the most common non-HNC referrals for MBS studies in a comprehensive cancer center, including neuro-oncology, endocrine/thyroid, thoracic cancers, and mixed non-HNC cancers in in- and outpatient practice settings. Key findings reveal that DIGEST maintains strong reliability, criterion validity, and construct validity in these populations and settings, with consistent performance across cancer subgroups and clinical settings.

DIGEST_v2_ demonstrated inter-rater reliability comparable to that reported in prior studies on HNC (k_w_=0.67–0.81) [1, 2, 7, 8, 34] and ALS (k = 0.94) [12] populations. The importance of this result lies in the fact that reliability was established for the first time using clinician-rated MBS from the health record, thus using real-world clinical data. This finding underscores the ecological validity of DIGEST, as it demonstrates the validity not only of research laboratory grading but also clinical reporting, thus supporting the tool’s practical utility. Unlike the initial validation studies, which relied exclusively on research-driven ratings, this study reinforces DIGEST’s robustness as a clinically adopted tool. Ongoing work (R01CA271223) will explore whether reliability is maintained under different clinical protocols and conditions, which if demonstrated would further strengthening DIGEST’s clinical utility in diverse populations and practice settings.

Criterion validity results were consistent with those observed in HNC (r_s_ = 0.77, *p* < 0.001) and ALS (F[3, 96] = 24.7, *p* < 0.001) [12], further supporting DIGEST’s ability to accurately assess pharyngeal dysphagia severity. Importantly, the ordinal nature of DIGEST grades was also confirmed, including for grade 4 as distinct from grade 3, which had not been previously established. This new evidence for the ability to differentiate more extreme dysphagia grades (severe versus profound/life threatening) expands the tool’s applicability to patients with high-grade dysphagia, a group that may require more intensive management and monitoring, thus improving the overall scope of its use.

Another important finding is the consistency of DIGEST’s performance across diverse oncology populations and clinical settings, as shown in the subgroup analysis. This consistency was observed across neuro-oncology, endocrine/thyroid, thoracic cancers, all of which have distinct treatment regimens and physiological mechanisms that can influence swallowing [14]. For example, patients with lung cancer may experience dysphagia due to respiratory compromise, recurrent laryngeal nerve compression or injury, or radiation-induced damage to surrounding structures, resulting in varied dysphagia manifestations [18, 22, 35, 36]. In contrast, those with CNS malignancies may experience dysphagia as a result of sensorimotor or cognitive impairments due to brain tumor progression or treatment-related side effects, leading to different dysphagia presentations unique to these underlying causes [19–21]. The fact that DIGEST maintained validity across these subgroups suggests that the tool is adaptable to assess dysphagia in a range of cancer types and manifestations, confirming its broad applicability. This consistency also strengthens the case for using DIGEST as a standardized tool for dysphagia assessment enterprise wide in cancer center settings.

This study has many strengths including representation across the continuum, robust statistical power for sub-group and between-grade analysis, and rigorous research laboratory grading methods to benchmark clinical DIGEST results. There are also limitations that warrant consideration. The purposeful sampling approach, designed to ensure equal representation across cancer subgroups, prevents the estimation of dysphagia prevalence from this dataset. However, this approach was essential to evaluate DIGEST’s psychometric properties with adequate statistical power across subgroups. Focusing on only three specific cancer subgroups may have overlooked findings in other cancer sub-populations. These subgroups were selected due to their relatively high dysphagia rates and frequent MBS referral. However, future research could focus on additional sub-populations, such as hematologic and gastrointestinal cancers, to further assess DIGEST applicability across the full spectrum of oncology settings and cancer types with varying physiologies and treatments. Finally, construct validity was assessed using HNC-specific measures (i.e., MDADI and PSS-HN). While these instruments allowed for direct comparisons with the original HNC validation study, they may not fully capture constructs relevant to non-HNC populations.

## Conclusion

While DIGEST is already widely used in the investigators’ full oncology clinical practice, this study is the first to investigate its psychometric properties across diverse oncology populations and settings, using clinician-rated MBS data. By demonstrating robust reliability, criterion validity, and construct validity, this study supports the implementation of DIGEST as an evidence-based, clinically feasible tool for dysphagia assessment in non-HNC oncology populations. These findings lay the groundwork for its broader adoption and equitable application across oncology care, ensuring improved outcomes for all cancer survivors affected by dysphagia.

## Supplementary Information

Below is the link to the electronic supplementary material.

Supplementary Material 1

## Data Availability

The investigators compiled a dataset for public sharing merging all the extant sub-studies from the DIGEST Implementation project into a singular dataset for parsimony. The dataset [37] is available for public access on FigShare repository at 10.6084/m9.figshare.28544234.v1.

## Declarations

### Summary Statement

DIGESTv2 is a reliable and valid clinician-rated tool for grading the severity of pharyngeal swallowing impairment observed during video-based imaging of swallowing, with strong performance across diverse cancer populations.
