# Supplementary material for: Expanding the Evidence-base for Clinician-Graded Dysphagia Using Dynamic Imaging Grade of Swallowing Toxicity (DIGEST): Validation Across Non-head and Neck Cancer Oncology Populations
Source: Dysphagia. 2026 Mar 18;41(2):307–15. doi: 10.1007/s00455-025-10875-7 (PMC13002114; doi:10.1007/s00455-025-10875-7)
Supplement: Supplementary file 1 — Supplementary Material 1 [file 455_2025_10875_MOESM1_ESM.pdf]

SUPPLEMENTARY INFORMATION

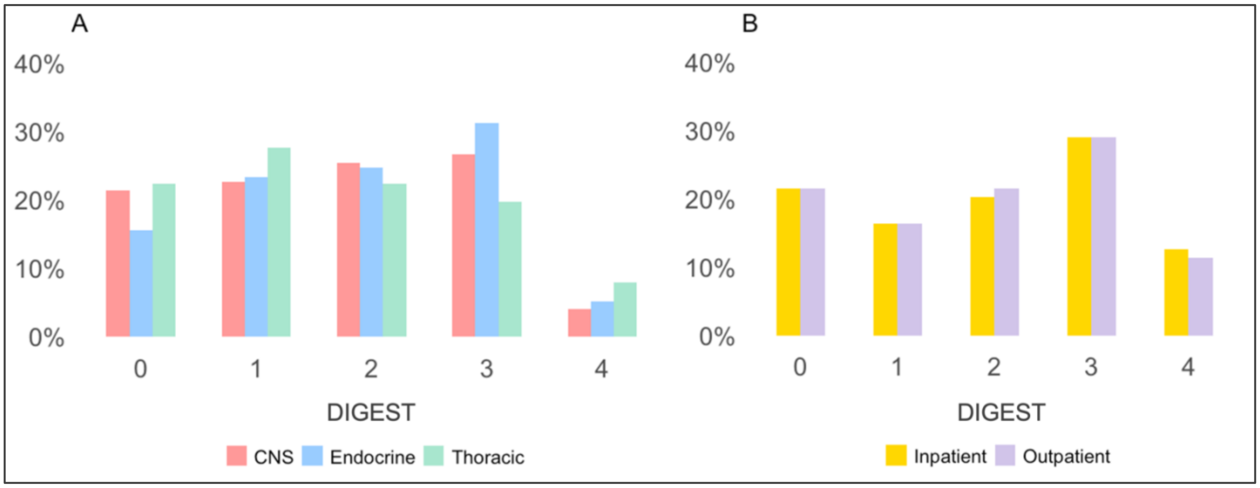

eFigure 1. Distribution of DIGEST grades by major disease sites (n=228, A) and practice settings (n=158, B). Abbreviations: CNS = central nervous system; DIGEST = Dynamic Imaging Grade of Swallowing Toxicity

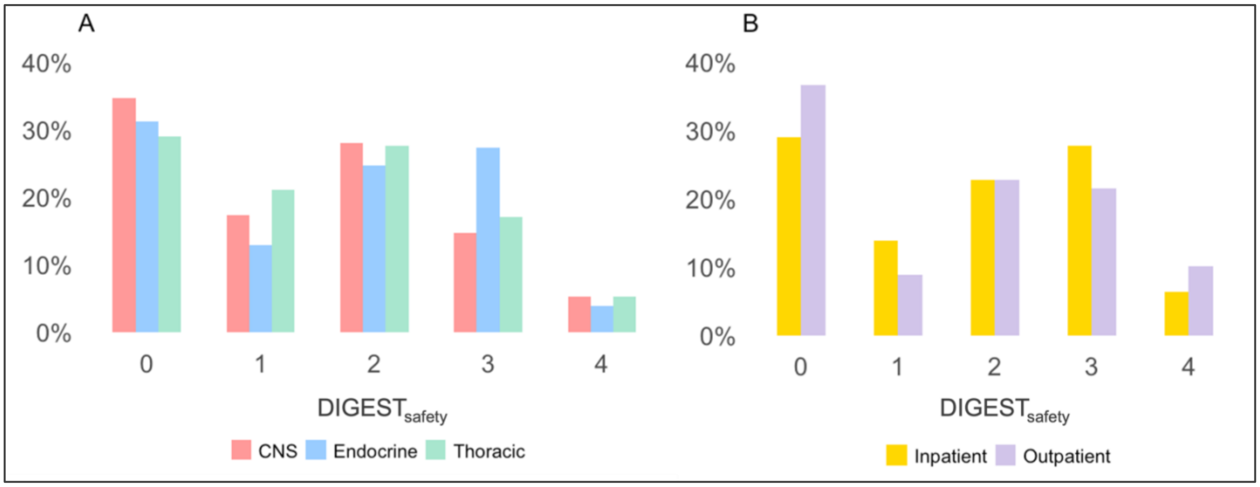

eFigure 2. Distribution of DIGEST-S grades by major disease sites (n=228, A) and practice settings (n=158, B). Abbreviations: CNS = central nervous system; DIGEST = Dynamic Imaging Grade of Swallowing Toxicity

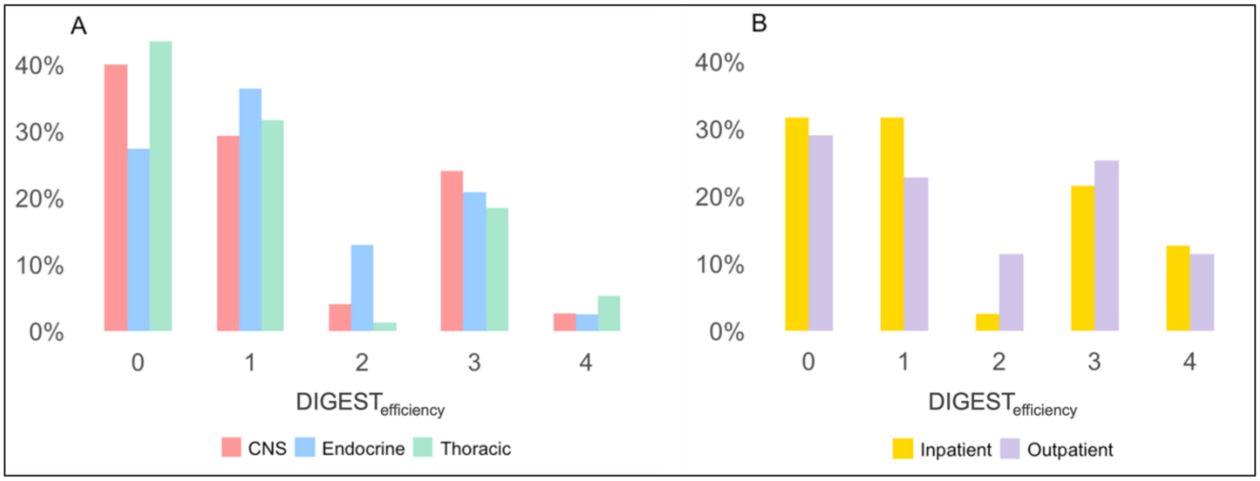

eFigure 3. Distribution of DIGEST-E grades by major disease sites (n=228, A) and practice settings (n=158, B). Abbreviations: CNS = central nervous system; DIGEST = Dynamic Imaging Grade of Swallowing Toxicity

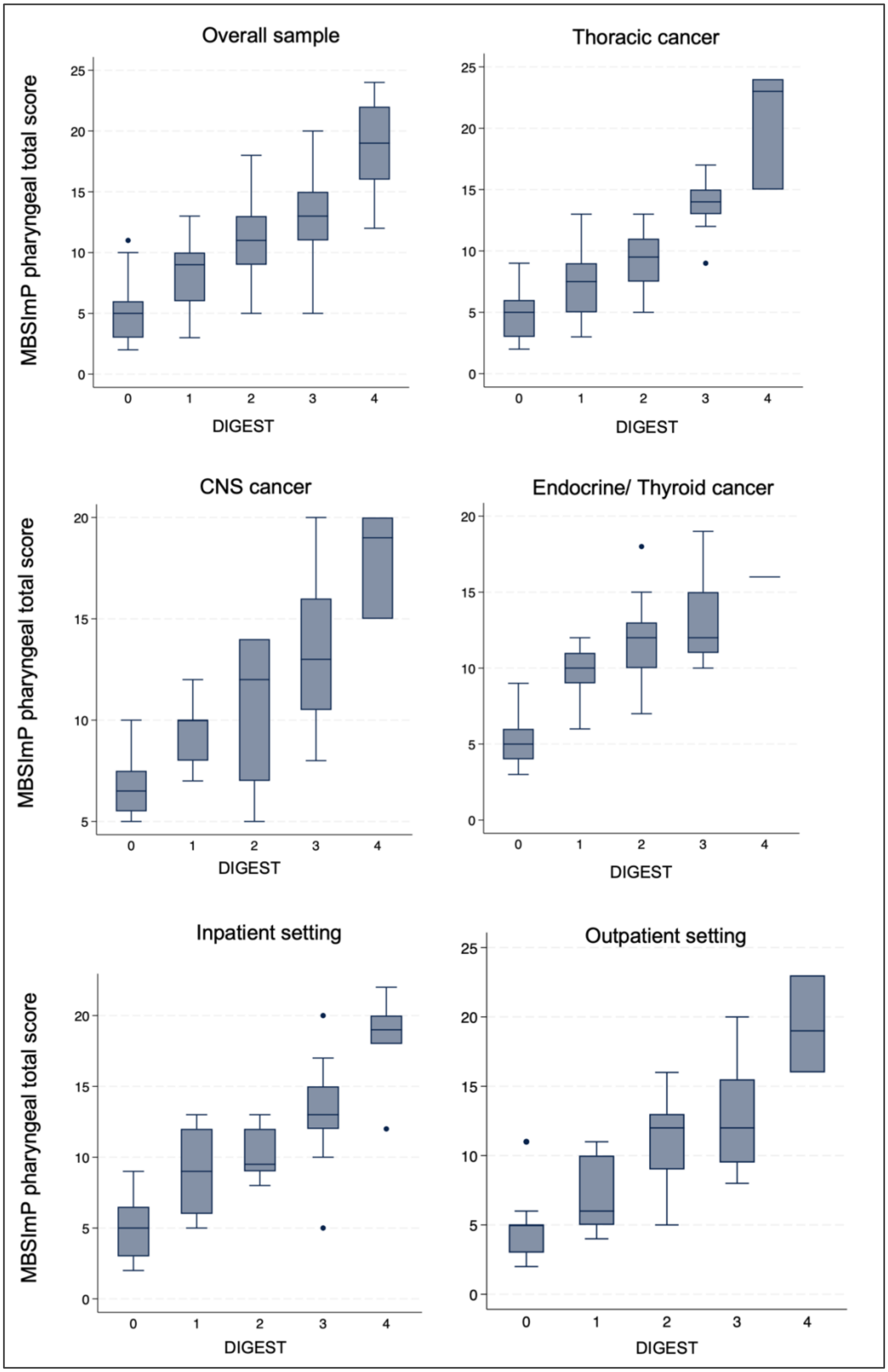

eFigure 4. Sensitivity analysis for DIGEST grade according to MBSImP pharyngeal total score *(including component 13: pharyngeal contraction in antero-posterior view)*. DIGEST distinguished levels of

pharyngeal pathophysiology for overall sample (n=309; r_s=0.78, 95% CI: 0.74-0.83), CNS cancer (n=55; r_s=0.75, 95% CI: 0.62-0.88), endocrine/ thyroid (n=71; r_s=0.74, 95% CI: 0.62-0.86), thoracic (n=57; r_s=0.77, 95% CI: 0.65-0.90), inpatient (n=54; r_s=0.81, 95% CI: 0.70-0.92) and outpatient (n=71; r_s=0.79, 95% CI: 0.69-0.89) subgroups. Scale ordinality for DIGEST grades in the total sample was also confirmed.

Abbreviations: CI = Confidence interval; CNS = central nervous system; DIGEST= Dynamic Imaging Grade of Swallowing Toxicity; MBSImP = Modified Barium Swallow Impairment Profile.
